# Correction: Direct Correlation between Motile Behavior and Protein Abundance in Single Cells

**DOI:** 10.1371/journal.pcbi.1005149

**Published:** 2016-09-30

**Authors:** The PLOS Computational Biology Staff

The corresponding authors of this paper should be listed as follows:

Yann S. Dufour, dufourya@msu.edu

Thierry Emonet, thierry.emonet@yale.edu

The Data Availability statement should read as follows:

The code used for single-cell tracking is available at https://github.com/dufourya/SwimTracker. The code used for the FAST experiment is available at https://github.com/ dufourya/FAST. The data are available publicly at https://dx.doi.org/10.6084/m9.figshare.3593046 or upon request by contacting the corresponding authors at dufourya@msu.edu and thierry.emonet@yale.edu.

The Acknowledgments section should read as follows:

We thank Adam Waite for improvements of the SwimTracker code, and Adam Waite and Setsu Kato for comments on the manuscript. We thank Victor Sourjik and Sandy Parkinson for their generous gifts of strains.

The publisher apologizes for these errors.
