# Supplementary material for: Direct Correlation between Motile Behavior and Protein Abundance in Single Cells
Source: PLoS Comput Biol. 2016 Sep 6;12(9):e1005041. doi: 10.1371/journal.pcbi.1005041 (PMC5012591; doi:10.1371/journal.pcbi.1005041)
Supplement: S1 Table — (DOCX) [file pcbi.1005041.s001.docx]

S1 Table. Plasmids.

| **Name** | **Origin of replication** | **Marker** | **Promoter** | **Gene(s) of interest** | **Description** | **Reference** |
| --- | --- | --- | --- | --- | --- | --- |
| pKD46 | repA101ts | AmpR | pAraB | *gam, bet, exo* | Temperature sensitive plasmid containing the Phage λ Red recombination system | (Datsenko & Wanner, 2000) |
| pCP15 | pMB1 | AmpR, KanR |  | *FRT-KanR-FRT* | Kanamycin resistance cassette flanked by FRT sequences | (Cherepanov & Wackernagel, 1995) |
| pCP16 | pMB1 | AmpR, TetR |  | *FRT-tetAR-FRT* | TetRacycline resistance cassette flanked by FRT sequences | (Cherepanov & Wackernagel, 1995) |
| pCP20 | repA101ts | AmpR, CmR |  | *flp* | Temperature sensitive plasmid expressing the FLP recombinase (“flippase”) | (Cherepanov & Wackernagel, 1995) |
| pTU136 | R6K | AmpR | pLac | *ssdsbA-mCherry* | Template for mCherry gene sequence | (Uehara, Dinh, & Bernhardt, 2009) |
| pYSD1003 | pMB1 | AmpR |  | *sfYFP* | Template for super-folder mYFP gene sequence | This study |
| pYSD1004 | pMB1 | AmpR |  | *sfCFP* | Template for super-folder mCFP gene sequence | This study |
| pYSD1011 | pMB1 | KanR | pBla | *sfCFP* | mCFP under the control of the *bla* promoter from pUC19 | This study |
| pYSD1007 | pMB1 | AmpR, KanR | pLac | *cheB-mYFP* | Template for the translational fusion of CheB and mYFP | This study |
| pYSD1005 | pMB1 | AmpR, KanR | pLac | *mCherry-cheR* | Template for the translational fusion of mCherry and CheR | This study |
