# Supplementary material for: Direct Correlation between Motile Behavior and Protein Abundance in Single Cells
Source: PLoS Comput Biol. 2016 Sep 6;12(9):e1005041. doi: 10.1371/journal.pcbi.1005041 (PMC5012591; doi:10.1371/journal.pcbi.1005041)
Supplement: S2 Table — (DOCX) [file pcbi.1005041.s002.docx]

S2 Table. Strains.

| **Name** | **Description** | **Reference** |
| --- | --- | --- |
| RP437 | Wild-type for chemotaxis | (Parkinson, 1978) |
| RP4972 | ΔcheB | (Parkinson, 1978) |
| NWF121 | ΔcheRcheB-FRT with ~300 bp CheB 3' fragment | This study |
| YSD2023 | pLac-mCherry-cheR, FRT-kanR-FRT | This study |
| YSD2024 | pRha-mCherry-cheR, FRT-kanR-FRT | This study |
| YSD2025 | pLac-cheB-mYFP, FRT-kanR-FRT | This study |
| YSD2027 | pRha-cheB-mYFP, FRT-kanR-FRT | This study |
| YSD2031 | pBla-mCFP, FRT-kanR-FRT | This study |
| YSD2040 | ΔcheRcheB, FRT, pLac-cheB-mYFP, FRT | This study |
| YSD2041 | ΔcheRcheB, FRT, pLac-mCherry-cheR, FRT | This study |
| YSD2044 | ΔcheB, pLac-cheB-mYFP, FRT | This study |
| YSD2062 | ΔcheRcheB, FRT, pLac-cheB-mYFP, FRT, pRha-mCherry-cheR, FRT | This study |
| YSD2063 | ΔcheRcheB, FRT, pRha-cheB-mYFP, FRT, pLac-mCherry-cheR, FRT | This study |
| YSD2072 | ΔcheRcheB, FRT, pLac-cheB-mYFP, FRT, pRha-mCherry-cheR, FRT, pBla-mCFP, FRT | This study |
| YSD2073 | ΔcheRcheB, FRT, pRha-cheB-mYFP, FRT, pLac-mCherry-cheR, FRT, pBla-mCFP, FRT | This study |
