# Supplementary material for: Direct Correlation between Motile Behavior and Protein Abundance in Single Cells
Source: PLoS Comput Biol. 2016 Sep 6;12(9):e1005041. doi: 10.1371/journal.pcbi.1005041 (PMC5012591; doi:10.1371/journal.pcbi.1005041)
Supplement: S3 Table — (DOCX) [file pcbi.1005041.s003.docx]

S3 Table. Oligonucleotides primer list.

| **Construct** | **Primer name** | **Sequence** | **Template** | **Product** |
| --- | --- | --- | --- | --- |
| **For Gibson assembly** | | | | |
| pYSD1011 | PUC19_PBLA_SYNTH_FOR | CATACTCTTCCTTTTTCAATATTATTG | pUC19 | Vector backbone |
|  | PUC19_BLA_SYNTH_REV | TAAGCATTGGTAACTGTCAGAC |  |  |
|  | PUC_PBLA_MYFP_N | CAATAATATTGAAAAAGGAAGAGTATGGTGAGCAAGGGCGAGGAG | pYSD1004 | mCFP |
|  | FRT_N - MYFP_C | CGGGTACCGAGCTCGAATTCTTACTTGTACAGCTCGTCCATGCC |  |  |
|  | FRT-N | GAATTCGAGCTCGGTACCCG | pCP15 | FRT-kanR-FRT |
|  | PUC_BLA_FRT_C | GTCTGACAGTTACCAATGCTTAAAGCTTCAAAAGCGCTCTGA |  |  |
| pYSD1007 | PUC19UNIV-SYNTHESISNONOT1 FOR | GATCCTCTAGAGTCGACCTG | pUC19 | Vector backbone |
|  | PUC19UNIV-SYNTHESISNONOT1 REV | CGGGTACCGAGCTCGAATTC |  |  |
|  | PUC19 - CHEB | CAGGTCGACTCTAGAGGATCATGAGCAAAATCAGGGTGTTATCTG | *E. coli* RP437 | cheB |
|  | LINKER_CHEB-C | GGAACCTCCACCGCCAATACGTATCGCCTGTCCGG |  |  |
|  | CHEB-C_LINKER_MYFP-N | CCGGACAGGCGATACGTATTGGCGGTGGAGGTTCCGTGAGCAAGGGCGAGGAG | pYSD1003 | mYFP |
|  | FRT_N - MYFP_C | CGGGTACCGAGCTCGAATTCTTACTTGTACAGCTCGTCCATGCC |  |  |
|  | FRT-N | GAATTCGAGCTCGGTACCCG | pCP15 | FRT-kanR-FRT |
|  | PUC19 - FRT | GAATTCGAGCTCGGTACCCGAAGCTTCAAAAGCGCTCTGA |  |  |
| pYSD1005 | PUC19UNIV-SYNTHESISNONOT1 FOR | GATCCTCTAGAGTCGACCTG | pUC19 | Vector backbone |
|  | PUC19UNIV-SYNTHESISNONOT1 REV | CGGGTACCGAGCTCGAATTC |  |  |
|  | PUC19 - MYFP | CAGGTCGACTCTAGAGGATCATGGTGAGCAAGGGCGAGGAG | pTU136 | mCherry |
|  | CHER-N_LINKER_MYFP-C | CCCACAGGGCAGAGATGAAGTGCCGCCGCCGCCGCCCTTGTACAGCTCGTCCATGCC |  |  |
|  | LINKER_CHER-N | GGCGGCGGCGGCGGCACTTCATCTCTGCCCTGTGGG | *E. coli* RP437 | cheR |
|  | FRT-N_CHER-C | CGGGTACCGAGCTCGAATTCTTAATCCTTACTTAGCGCATACAC |  |  |
|  | FRT-N | GAATTCGAGCTCGGTACCCG | pCP15 | FRT-kanR-FRT |
|  | PUC19 - FRT | GAATTCGAGCTCGGTACCCGAAGCTTCAAAAGCGCTCTGA |  |  |
| **For Lambda Red recombination** | | | | |
| NWF121 | CHER_TETF | CATGAAGTAGCACGACATGAGTCGGTGCAGTTACAAATTGCGCCAGTGGTATCCTGAAGTGATTGAGAAGGCGCTATGACTTCATCTCTGTGCCACCTGACGTCTAAGAA | pCP16 | FRT-tetAR-FRT |
|  | CHEBALT_TETR | GTTAACCGCCGGGCCATCGTGAATTTTGATTTGGTAATTTGCGCCACTACGCGACAGCTCCATATGCCGATCGCCCGGCGCAATATAGGCTTTGCGCATTCACAGTTCTC |  |  |
| YSD2023 | PLAC-MYFP | TATGTTGTGTGGAATTGTGAGCGGATAACAATTTCACACAGGAAACAGCTATGGTGAGCAAGGGCGAGGAG | pYSD1005 | pLac-mCherry-CheR-FRT-kanR-FRT-lacZ’ |
|  | LACZ-C_FRT | TTATTTTTGACACCAGACCAACTGGTAATGGTAGCGACCGGCGCTCAGCTAAGCTTCAAAAGCGCTCTGA |  |  |
| YSD2024 | PRHA-MYFP | ATTCAGGCGCTTTTTAGACTGGTCGTAATGAAATTCAGCAGGATCACATTATGGTGAGCAAGGGCGAGGAG | pYSD1005 | pRha-mCherry-CheR-FRT-kanR-FRT-rhaA’ |
|  | RHAA-C_FRT | TTACCCGCGGCGACTCAAAATTTCTTTCTCATAAGCCCGCACGCTCTCCAAAGCTTCAAAAGCGCTCTGA |  |  |
| YSD2025 | PLAC-CHEB | TATGTTGTGTGGAATTGTGAGCGGATAACAATTTCACACAGGAAACAGCTATGAGCAAAATCAGGGTGTTATCTG | pYSD1007 | pLac-CheB-mYFP-FRT-kanR-FRT-lacZ’ |
|  | LACZ-C_FRT | TTATTTTTGACACCAGACCAACTGGTAATGGTAGCGACCGGCGCTCAGCTAAGCTTCAAAAGCGCTCTGA |  |  |
| YSD2027 | PRHA-CHEB | ATTCAGGCGCTTTTTAGACTGGTCGTAATGAAATTCAGCAGGATCACATTATGAGCAAAATCAGGGTGTTATCTG | pYSD1007 | pRha-CheB-mYFP-FRT-kanR-FRT-rhaA’ |
|  | RHAA-C_FRT | TTACCCGCGGCGACTCAAAATTTCTTTCTCATAAGCCCGCACGCTCTCCAAAGCTTCAAAAGCGCTCTGA |  |  |
| YSD2031 | ARAA_PUC_PBLA | TAGCGACGAAACCCGTAATACACTTCGTTCCAGCGCAGCGCGTCTTTAAATCTCAGTACAATCTGCTCTGA | pYSD1011 | araA’-pBla-mCFP-FRT-kanR-FRT-araB’ |
|  | ARAB_PUC_BLA | GGCGATGAGCGCCGAACAACACTATCTTCCAACTTCCGCCCCGGCACAGGAACTTGGTCTGACAGTTACCA |  |  |
