# Supplementary material for: Direct Correlation between Motile Behavior and Protein Abundance in Single Cells
Source: PLoS Comput Biol. 2016 Sep 6;12(9):e1005041. doi: 10.1371/journal.pcbi.1005041 (PMC5012591; doi:10.1371/journal.pcbi.1005041)
Supplement: S4 Table — (DOCX) [file pcbi.1005041.s004.docx]

S4 Table. Model parameters

| **Receptor Parameters** | | | | | |
| --- | --- | --- | --- | --- | --- |
| **Name** | **Description** | | **Value** | **Reference** | |
| *ε*_1_ | Receptor energy change per methyl group addition | | -1 k_B_T | Shimizu et al. 2010 | |
| **Signaling Parameters** | | | | | |
| **Name** | | **Description** | **Value** | **Reference** | |
| *k_r_* | | Catalytic rate of CheR | 0.42 s^-1^ | Frankel et al., 2014 | |
| *K_r_* | | Equilibrium constant of CheR activity | 1200 μM | Frankel et al., 2014 | |
| *k_b_* | | Catalytic rate of CheB demethylation | 0.32 s^-1^ | Frankel et al., 2014 | |
| *k_Q_* | | Catalytic rate of CheB deamination | 0.64 s^-1^ | *this study* | |
| *K_b_* | | Equilibrium constant of CheB activity | 800 μM | Frankel et al., 2014 | |
| *a_P_* | | CheA autophosphorylation rate | 12.5 s^-1^ | Frankel et al., 2014 | |
| *a_B_* | | Rate of CheB phosphorylation by CheA | 15 μM^-1^ s^-1^ | Stewart, Jahreis, and Parkinson, 2000 | |
| *d_B_* | | CheB autodephosphorylation rate | 0.5 s^-1^ | Stewart, 1993, Kentner and Sourjik, 2006 | |
| *a_Y_* | | Rate of CheY phosphorylation by CheA | 50 μM^-1^ s^-1^ | Frankel et al., 2014 | |
| *d_Z_* | | Rate of CheY desphosphorylation by CheZ | 5 μM^-1^ s^-1^ | Frankel et al., 2014 | |
| **Motor Parameters** | | | | | |
| **Name** | | **Description** | **Value** | **Reference** | |
| *ω*_0_ | | Basal switching frequency | 1.3 s^-1^ | Sneddon et al., 2012, Cluzel et al., 2000 | |
| *ε_3,0_* | | Motor steepness | 80 | Yuan et al., 2013, Dufour et al., 2014 | |
| *K_D_* | | Dissociation constant of CheY-motor interaction | 3.06 μM | Sneddon et al., 2012, Cluzel et al., 2000 | |
| *k­_on_* | | Rate of motor adaptation | 0.025 s^-1^ | Dufour et al., 2014 | |
| *ε_3,1_* | | Slope of motor steepness response to change in bound FliM | 1.96 | Dufour et al., 2014 | |
| *∆_n_* | | Effective half-max of FliM binding to the motor | 4.16 | Dufour et al., 2014 | |
| *n_0_* | | Number of FliM on the motor at rest | 36 |  | |
| *n_1_* | | Minimum number of FliM on the motor | 34 | Dufour et al., 2014 | |
| *n_2_* | | Maximum number of FliM on the motor | 44 | Dufour et al., 2014 | |
| **Flagellar bundle parameters** | | | | | |
| **Name** | | **Description** | **Value** | **Reference** | |
| *λ* | | Mean waiting time of semi-coiled to curly transition | 0.2 s | Sneddon et al., 2012 | |
| *N_flagella_* | | Total number of flagella per cell | 4 | Sneddon et al., 2012 | |
| *N_bundle_* | | Number of flagella rotating CCW to form a bundle | 2 | Sneddon et al., 2012 | |
| **Gene expression parameters** | | | | | |
| **Name** | | **Description** | **Value** | **Reference** | |
| *T_Tot_* | | Population mean receptors per cell (Tar + Tsr) | 26000 mol./cell | Li and Hazelbauer, 2004 | |
| *A_Tot_* | | Population mean CheA proteins per cell | 7700 mol./cell | Li and Hazelbauer, 2004 | |
| *W_Tot_* | | Population mean CheW proteins per cell | 7200 mol./cell | Li and Hazelbauer, 2004 | |
| *R_Tot_* | | Population mean CheR proteins per cell | 160 mol./cell | Li and Hazelbauer, 2004 | |
| *B_Tot_* | | Population mean CheB proteins per cell | 270 mol./cell | Li and Hazelbauer, 2004 | |
| *Y_Tot_* | | Population mean CheY proteins per cell | 6300 mol./cell | Li and Hazelbauer, 2004 | |
| *Z_Tot_* | | Population mean CheZ proteins per cell | 2700 mol./cell | Li and Hazelbauer, 2004 | |
| *x* | | Conversion between mol./cell and mM for proteins | 833 μM/(mol./cell) | Frankel et al., 2014 | |
| *A_YZ_* | | Translational coupling coefficient between CheY and CheZ | 0.25 | Lovdok et al., 2009 | |
| *η* | | Intrinsic noise scaling coefficient | 0.0125 | *this study* | |
| *ω* | | Extrinsic noise scaling coefficient | 0.026 | *this study* | |
| **Cell growth parameter** | | | | | |
| **Name** | | **Description** | **Value** | | **Reference** |
| *r* | | Cell generation time | 1 h^-1^ | | *this study* |
